# Inactivated Enterovirus 71 Particle Aggregation Stability: Dynamic Light Scattering Analysis and Stabilizer Identification

**DOI:** 10.3390/vaccines13121247

**Published:** 2025-12-15

**Authors:** Anna Yang, Dongsheng Yang, Deqin Pang, Jie Yang, Wenhui Wang, Yaxin Du, Xin Wan, Shengli Meng, Jing Guo, Shuo Shen

**Affiliations:** 1 Wuhan Institute of Biological Products Co., Ltd., No. 1 Huangjin Industrial Park Road, Wuhan 430207, China; 2 National Engineering Technology Research Center of Combined Vaccines, No. 1 Huangjin Industrial Park Road, Wuhan 430207, China; 3 State Key Laboratory of Novel Vaccines for Emerging Infectious Diseases, No. 1 Huangjin Industrial Park Road, Wuhan 430207, China; 4 Hubei Provincial Vaccines Technology Innovation Center, No. 1 Huangjin Industrial Park Road, Wuhan 430207, China

**Keywords:** inactivated coxsackievirus 71 vaccine, dynamic light scattering, particle size, protein aggregation, formulation development

## Abstract

Background: Inactivated enterovirus 71 (EV71) vaccines play a vital role in preventing severe cases of hand, foot, and mouth disease, with their quality and stability determined by the degree of viral particle aggregation. Objective: This study aimed to use dynamic light scattering (DLS) for monitoring EV71 particle size, comprehensively evaluate the effects of environmental stresses on viral aggregation, and identify suitable stabilizing agents. Methods: The DLS technique was validated. Using this method, the effects of pH, ionic strength, freeze–thaw cycles, temperature, and mechanical stresses on viral particle size were assessed. Additionally, the ability of different buffer salts and stabilizers to inhibit stress-induced aggregation was systematically evaluated. Results: The DLS method exhibited robust performance. EV71 particles were stable at pH 7.0–7.5. Exposure to 47 °C and magnetic stirring promoted viral aggregation. Phosphate buffer and citrate buffer exhibited the highest inhibitory effects on heat-induced aggregation and stirring-induced aggregation, respectively. M199 and Tween 80 efficiently mitigated heat-induced particle aggregation and shear stress-induced particle aggregation, respectively. Conclusions: This study demonstrated the performance of DLS in viral aggregation monitoring. Additionally, this study revealed tailored stabilization strategies, providing key insights for vaccine formulation and quality control.

## 1. Introduction

Hand, foot, and mouth disease (HFMD) and herpangina (HA), which are acute infectious diseases primarily caused by coxsackieviruses and enteroviruses, are highly contagious and predominantly affect children aged < 5 years [1,2]. The typical clinical manifestations of HFMD include oral herpes/ulcers and rashes on the hands, feet, and buttocks. Severe cases can lead to neurological complications (such as brainstem encephalitis and aseptic meningitis) and even death [3,4]. EV71 is a major causative agent for severe clinical manifestations and fatality [5,6]. Currently, specific antiviral drugs for EV71 infections are not available. Patients with EV71 infections are primarily managed through supportive treatment. The successful development, licensure, and widespread administration of the inactivated EV71 vaccine have effectively decreased the incidence of EV71-associated HFMD, especially severe disease and mortality, representing a milestone achievement in public health prevention and control [7,8,9,10].

The inactivated EV71 vaccine belongs to the group of traditional viral particle vaccines. Similar to that of other conventional prophylactic vaccines (such as inactivated and live attenuated vaccines), the composition of the inactivated EV71 vaccine is complex [11]. In particular, inactivated EV71 vaccines comprise intact viral particles, potential subunit components, process-related impurities, and various excipients. The structural integrity and particulate homogeneity are crucial for ensuring immunogenicity of the vaccine. Nevertheless, the complex composition of vaccines presents a major challenge for comprehensive quality assessment [12]. Conventional quality control approaches primarily evaluate purity (including residual host cell proteins and DNA), physicochemical parameters (such as pH and osmolality), sterility, and critical biological potency indicators (including immunogenicity and in vivo potency). However, systematic characterization and monitoring at the microscopic particle level—particularly concerning viral particle aggregation—are rarely implemented. Aggregation of viral particles can occur due to intrinsic surface characteristics (such as hydrophobic regions and charge distribution) and extrinsic factors (including pH, ionic strength, temperature, and mechanical stress) [13,14,15,16]. Throughout vaccine manufacturing, purification, inactivation, formulation, storage, and transport, viral particles may undergo aggregation, producing aggregates of varying sizes [17,18,19]. The formation of antigen aggregates can alter vaccine immunogenicity, impair the induction of effective neutralizing antibodies, and potentially increase risks, such as triggering unexpected or enhanced adverse reactions. Additionally, aggregation is accompanied by a decline in physical stability, potentially leading to potency loss, which directly impacts vaccine efficacy and shelf-life [20,21]. Therefore, understanding and effective control of viral particle aggregation behavior is essential to ensure the quality, safety, and efficacy of inactivated EV71 vaccines and other traditional viral particle vaccines. While biological potency is the ultimate quality attribute, the development of robust vaccines relies on efficient in vitro tools to monitor critical quality attributes during early development. Physicochemical methods, such as DLS, serve as rapid, sensitive proxies for physical stability, enabling high-throughput screening of formulations before committing resources to confirmatory biological assays.

Dynamic light scattering (DLS) is a well-established, non-invasive technique to measure the size and size distribution of molecules and particles in the sub-micron range. In DLS, the Brownian motion of particles in solution is measured to determine the hydrodynamic diameter (Z-avg). Thus, DLS has applications in rapidly assessing the average size of protein aggregates and sample stability [22,23,24]. Furthermore, techniques like DLS and nanoparticle tracking analysis (NTA) have been successfully applied to characterize the stability and particle size distribution of various viral vaccines, including inactivated rabies and other viral particles [25,26]. underscoring their utility in the comprehensive quality assessment of complex biologics. Moreover, recent advancements in DLS methodologies, such as multi-angle detection, have expanded its utility to include the direct measurement of nanoparticle number concentration, offering a more comprehensive characterization beyond size and polydispersity [27,28].

This study comprehensively examined the effects of multiple environmental factors on the aggregation behavior of inactivated EV71 viral particles and assessed the protective efficacy of various stabilizing agents. The outcomes of this work provide a scientific foundation for elucidating the stability characteristics of inactivated EV71 vaccines and guiding the optimization of their manufacturing process and formulation design.

## 2. Materials and Methods

### 2.1. Chemicals and Reagents

The following reagents were obtained from different suppliers: Dulbecco’s modified Eagle’s medium (Gibco, Waltham, MA, USA); M199 medium (Gibco, Waltham, MA, USA); newborn calf serum (Runsun Great Cause, Datong, China); sodium hydroxide, hydrochloric acid, disodium hydrogen phosphate, sodium dihydrogen phosphate, citric acid, histidine, magnesium chloride (MgCl_2_), and trehalose (Sinopharm Chemical Reagent Co., Ltd., Shanghai, China); Tween 80 (Well Pharmaceutical Co., Ltd., Nanjing, China); and standard particles (Thermo Scientific, Waltham, MA, USA).

### 2.2. Preparation of Inactivated EV71 Bulk

The inactivated EV71 bulk was produced by Wuhan Institute of Biological Products Co., Ltd. (Wuhan, China). The manufacturing process of the inactivated viral particles involved several steps, including the revival and expansion of Vero cells (sourced from the WIBP cell bank), viral inoculation, virus propagation and harvesting, removal of cell debris, microfiltration, ultrafiltration, gel filtration chromatography, ion-exchange chromatography, formaldehyde inactivation, and desalination. Detailed information regarding the purification process of the vaccine bulk is proprietary to Wuhan Institute of Biological Products Co., Ltd. and cannot be disclosed.

### 2.3. Sample Preparation for Viral Particle Stability Analysis

To evaluate the influence of pH on viral particle size, the pH of the EV71 bulk (protein concentration: 80 μg/mL) was adjusted to 5.0, 5.5, 6.0, 6.5, 7.0, 7.5, and 8.0 using 0.1 M sodium hydroxide or 0.1 M hydrochloric acid. The effect of ionic strength on viral particle size was examined by adding sodium chloride to the EV71 bulk to achieve final concentrations of 150, 300, and 600 mM. For temperature stability assessment, samples of the EV71 bulk (80 μg/mL) were placed in a pharmaceutical stability chamber (YSEI, Chongqing, China) set at 25 °C, 37 °C, and 47 °C, or in a refrigerator (Haier, Qingdao, China) maintained at 4 °C.

The impact of mechanical agitation was tested by subjecting the EV71 bulk (80 μg/mL) to vortex mixing using a mixer (Eppendorf, Hamburg, Germany) at 300, 600, and 1200 rpm. A stirring stress test was conducted by placing the sample on a magnetic stirrer (ISMART, Espoo, Finland) with a stir bar rotating at the same speeds. To assess freeze–thaw stability, the EV71 bulk (80 μg/mL) underwent multiple freeze–thaw cycles, each consisting of freezing at −20 °C (Haier, Qingdao, China) for at least 24 h, followed by thawing at 4 °C (Haier, Qingdao, China) for over 24 h.

The effect of different buffering systems—phosphate (10 mM), citrate (20 mM), and histidine (20 mM)—on viral particle size was also evaluated. The EV71 bulk was mixed with each buffer at a 1:1 volume ratio, resulting in a final protein concentration of 40 μg/mL. The mixtures were then subjected to various stress conditions before particle size measurement. To determine the influence of stabilizers, solutions containing M199 (2% and 10% *w*/*v*), Tween 80 (0.02% and 0.10% *w*/*v*), and MgCl2 (50 and 100 mM) were prepared. Each stabilizer solution was combined with the EV71 bulk at a 1:1 volume ratio (final protein concentration: 40 μg/mL), and the resulting mixtures were exposed to accelerated stress conditions before size analysis.

### 2.4. Particle Size Measurement

The particle size distribution of the viral particles was analyzed using dynamic light scattering (DLS) with a Zetasizer Nano ZS90 instrument (Malvern Panalytical, Malvern, UK). After equilibrating at 25 °C for 30 min, 1 mL of each sample was transferred into a quartz cuvette (10 mm path length). Measurements were conducted at 25.0 °C following a 120 s equilibration period. The viscosity value for PBS at 25 °C (0.8882 cP) was used for all data processing. Each sample was measured in triplicate, with three independent runs of 90 s each. Correlation data were processed using DTS Nano Software (v7.13) to obtain the intensity-weighted mean hydrodynamic diameter (Z-average) and polydispersity index (PDI). For each stress condition, a sample was prepared, and its particle size was determined from three technical replicate measurements.

### 2.5. Transmission Electron Microscopy (TEM)

A 20 μL aliquot of the inactivated EV71 sample was adsorbed onto a carbon-coated copper grid (200 mesh) for 10 min. The grid was negatively stained with 20 μL of 1% phosphotungstic acid (pH 7.0) for 10 min. After the excess liquid was blotted off, the grid was air-dried. The morphological characteristics of the particles were examined via transmission electron microscopy (HITACHI, Tokyo, Japan).

## 3. Results

### 3.1. Validation of DLS for Size Analysis of Inactivated EV71 Viral Particles

The aggregation behavior of inactivated EV71 particles under different environmental conditions was analyzed using DLS to determine their hydrodynamic diameter. To verify the reliability of this technique, a systematic validation of its applicability was conducted.

The instrument performance was controlled using standard particles of known nominal diameters (20 ± 2 nm and 41 ± 1 nm). The DLS analysis yielded measured sizes of 24.1 ± 0.4 nm (*n* = 3) and 42.5 ± 0.4 nm (*n* = 3) (Supplementary Table S1), which aligned closely with the reference values. These results confirmed the accuracy of the instrument configuration and the suitability of the method for size characterization.

To establish the optimal protein concentration for EV71 particle measurement, purified samples were diluted with phosphate buffer to concentrations ranging from 10 to 80 μg/mL. The coefficient of variation (CV) in particle size was relatively high at 10 μg/mL (Supplementary Table S2), whereas it stabilized at concentrations of 20 μg/mL and above. Consequently, a concentration of higher than 20 μg/mL was selected for subsequent analyses.

**Figure 1:**
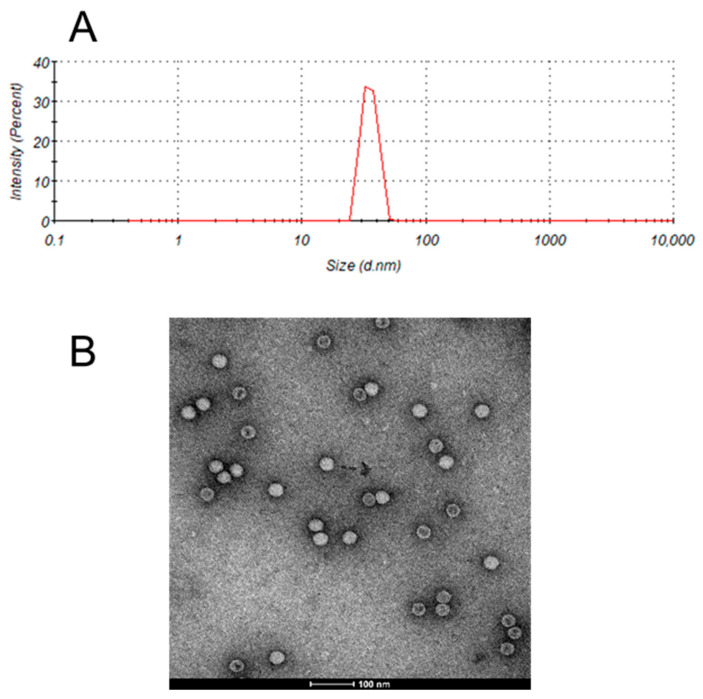
Particle size analysis of enterovirus 71 (EV71). (**A**) Size of the EV71 bulk determined using dynamic light scattering (DLS). (**B**) Transmission electron micrograph of the EV71 bulk.

The average hydrodynamic diameter of the inactivated EV71 bulk was approximately 35 nm (Figure 1A), consistent with the measurements obtained through transmission electron microscopy (Figure 1B). Thus, the particle size range of EV71 viral particles was validated by two independent analytical methods.

The precision of the DLS method was further assessed. The CV for six consecutive measurements of the same sample was 1.85% (Supplementary Table S3), while that for three independent measurements performed on separate days was 1.01% (Supplementary Table S4). These results demonstrated the high repeatability and precision of the DLS technique.

In summary, the outcomes of instrument calibration using standards, optimization of concentration range, cross-validation with another analytical method, and precision testing confirmed that DLS is a reliable approach for determining particle size and monitoring the stability of inactivated EV71 viral particles.

### 3.2. Stability Assessment of EV71 Viral Particles Based on DLS

This study systematically evaluated the size stability of EV71 viral particles under various environmental stress conditions using DLS, providing critical insights for vaccine formulation and process development.

**Figure 2:**
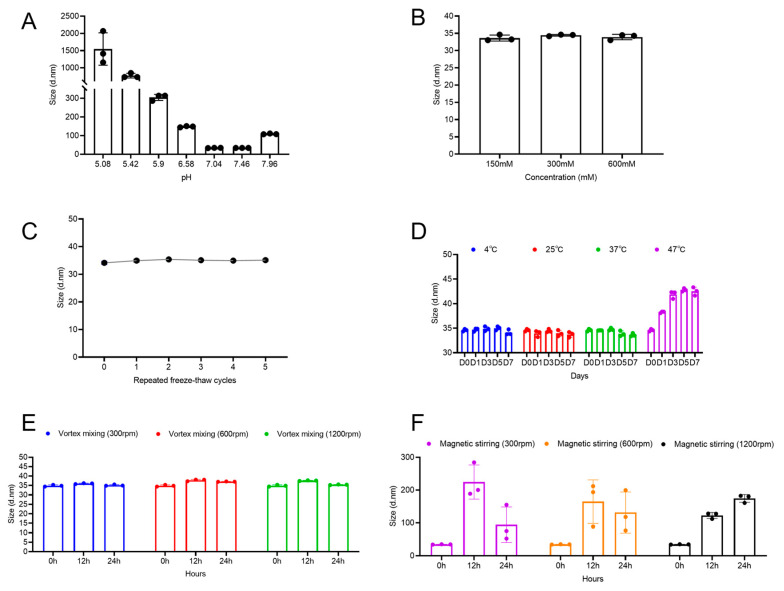
Effects of different conditions on enterovirus 71 (EV71) viral particle size. (**A**) Particle size of EV71 bulk at different pH values, (**B**) different sodium chloride concentrations, (**C**) after 1–5 freeze-thaw cycles, (**D**) at different temperatures, (**E**) after vortex mixing, and (**F**) after magnetic stirring.

The influence of pH on the stability of viral particles was examined by adjusting the pH of the EV71 bulk solution between 5.0 and 8.0, followed by measurement of the hydrodynamic diameter. As shown in Figure 2A, particle size remained consistent at approximately 34 nm within the pH range of 7.0–7.5. However, a significant increase in size was observed when the pH exceeded 8.0 or fell below 6.5. Imaged capillary isoelectric focusing analysis determined the isoelectric point (pI) of the viral particles to be 5.0–6.0, suggesting that aggregation occurred when the surrounding pH approached the pI, likely due to charge neutralization and diminished electrostatic repulsion. The observed aggregation at pH > 8.0 may be explained by the surface charge properties of the viral particles. With a pI of 5.0–6.0, the particles possess a strong net negative charge at neutral pH, ensuring colloidal stability via electrostatic repulsion. At alkaline pH, further deprotonation of surface groups reduces the net charge density, lowering the repulsive energy barrier and enabling aggregation driven by attractive intermolecular forces. Therefore, the optimal pH range for maintaining EV71 particle stability was identified as 7.0–7.5.

Subsequently, the effect of ionic strength on particle stability was analyzed. The particle size remained stable at approximately 34 nm across the tested sodium chloride concentrations (Figure 2B), indicating that ionic strength did not exert a notable influence on particle stability under these conditions.

Repeated freeze-thaw stress may occur during production and storage. Thus, this study subjected the samples to up to five freeze-thaw cycles (−20 °C/4 °C). The hydrodynamic diameter did not significantly vary throughout the testing period (Figure 2C). This indicates that EV71 viral particles exhibit good tolerance to repeated freeze-thaw stress without major irreversible aggregation.

The long-term effect of temperature on particle stability was also examined. The vaccine bulk was stored at 4 °C, 25 °C, 37 °C, and 47 °C, and the particle size was monitored over one week. As shown in Figure 2D, the particle size remained stable after 7 days of storage at 4 °C, 25 °C, and 37 °C, demonstrating good physical stability under these conditions. In contrast, under the accelerated stress condition of 47 °C, the particle size evolution exhibited a distinct two-phase profile. In the initial phase (Day 0 to Day 3), the size increased rapidly with increasing incubation time. In the following phase (Day 3 to Day 7), the particle size subsequently plateaued and stabilized at approximately 42 nm, suggesting the establishment of an equilibrium state. This phenomenon indicates that the aggregation process induced by elevated temperature was largely complete in the initial stage, resulting in a relatively stable mixture comprising native particles, oligomers, and aggregates of limited size. Notably, the final stabilized particle size (42 nm) slightly increased from the initial size (~35 nm), and the system maintained moderate polydispersity (PDI ~0.3). These observations suggest that the primary effect of 47 °C stress may be particle swelling or the formation of small, limited aggregates rather than extensive, uncontrolled aggregation.

Subsequently, the influence of vortex mixing and magnetic stirring on particle aggregation was examined. The two agitation methods produced distinctly different results. Across the tested speed range, vortex mixing did not significantly change the mean particle size of EV71, which remained stable at 34–37 nm (Figure 2E). This finding indicates that the shear forces generated by vortex mixing were insufficient to induce aggregation and may even have dissociated weak, preexisting aggregates. In stark contrast, magnetic stirring provoked a substantial increase in the mean particle size, which exceeded 200 nm (Figure 2F). This pronounced aggregation is likely attributable to the intense, localized shear forces and turbulent eddies introduced by the stir bar, which significantly increase the frequency and energy of interparticle collisions. The transient decrease in the mean particle size observed at 300 and 600 rpm between 12 and 24 h may indicate a reorganization of the aggregates into more compact structures or sedimentation of the largest aggregates out of the detection volume.

### 3.3. Screening of Stabilization Systems

Stability assessments indicated that elevated temperature (47 °C) and magnetic stirring markedly promoted the aggregation of inactivated EV71 viral particles. To examine the inhibitory effects of various buffer salts on aggregation under thermal and mechanical stress, the original sample buffer systems were substituted with commonly used buffer solutions—phosphate, citrate, and histidine—each adjusted to pH 7.2. The samples were subsequently incubated at 47 °C for seven days or exposed to magnetic stirring, followed by measurement of particle size.

**Figure 3:**
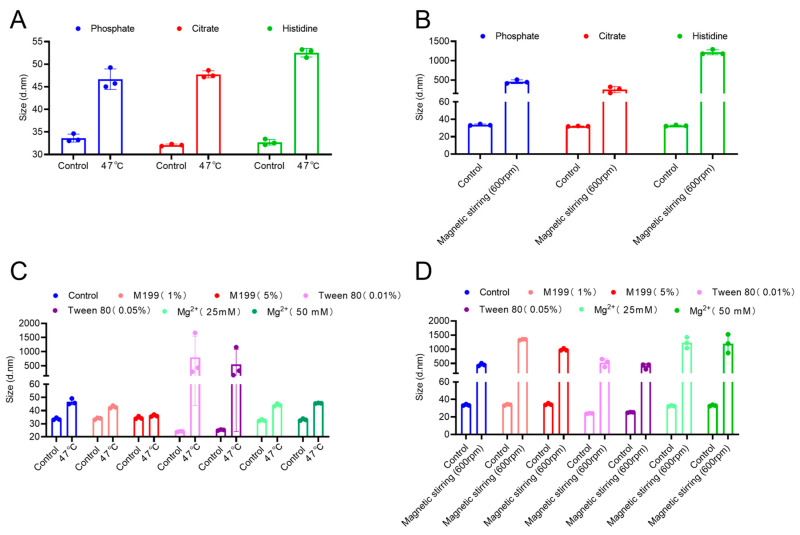
Screening of stabilization systems. (**A**) Particle size of enterovirus 71 (EV71) bulk in different buffer systems after heat treatment. (**B**) Particle size of EV71 bulk in different buffer systems after magnetic stirring. (**C**) Particle size of EV71 bulk with different stabilizers after heat treatment. (**D**) Particle size of EV71 bulk with different stabilizers after magnetic stirring.

As shown in Figure 3A,B, different buffer systems did not significantly affect the particle size in the absence of external stress. Under thermal stress conditions, all samples exhibited increased size. This indicates that heat promotes aggregation. In particular, the histidine buffer system promoted the highest aggregation, whereas the phosphate buffer system was associated with the smallest size increase, suggesting enhanced resistance to heat-induced aggregation. Magnetic stirring differentially increased the particle size in different buffer systems. The histidine system was associated with the highest increase in size. In contrast, the citrate system exerted the highest inhibitory effect on stirring-induced aggregation.

This study also evaluated the protective effects of several commonly used stabilizers (M199, Tween 80, and MgCl_2_). The bulk was incubated with different concentrations of these stabilizers and subjected to the thermal and stirring stability tests. The results (Figure 3C,D) revealed that under non-stress conditions, Tween 80 slightly reduced the initial particle size of EV71, potentially because of its dispersing action, which disrupted preexisting small aggregates in the sample. In contrast, M199 and magnesium chloride (MgCl_2_) had no significant effects. In the thermal stability test, M199 significantly inhibited the heat-induced increase in particle size, with its efficacy being concentration dependent. A 5% M199 concentration effectively maintained particle size stability even after exposure to 47 °C. These findings suggest that M199, as a complex culture medium, may maintain the conformational integrity of the viral capsid by providing a multidimensional stabilizing environment, including suitable osmotic pressure and a rich array of amino acids, to suppress protein denaturation. However, under the intense shear stress induced by magnetic stirring, the protective effects of all stabilizers were incomplete, as evidenced by a significant increase in particle size across all test groups. Nonetheless, the Tween 80-treated group presented the smallest increase in particle size. It is postulated that Tween 80 mitigates the aggregation process—driven by stirring-induced vortices and local interfacial denaturation—through the formation of a protective layer at the air-liquid interface via competitive adsorption. Nevertheless, the extreme physical disruptive forces generated by magnetic stirring are likely to exceed the protective capacity of conventional stabilizers.

## 4. Discussion

The prevention and management of HFMD and its severe complications rely on the effectiveness and safety of inactivated EV71 vaccines. Unlike conventional small-molecule drugs, viral particle vaccines possess a highly complex structure. The aggregation behavior of viral particles has a direct influence on their quality characteristics and safety profile.

In this study, DLS was employed to monitor the aggregation behavior of EV71 virus particles under various stress conditions. Importantly, the DLS-derived Z-average is an intensity-weighted mean diameter, which is highly sensitive to the presence of large aggregates in polydisperse systems. While this property makes DLS an excellent tool for detecting the onset of aggregation, as demonstrated in our study, the Z-average should be interpreted in conjunction with the PDI. The concurrent increase in both the Z-average and PDI under stresses such as elevated temperature and magnetic stirring unequivocally indicated not only particle growth but also broadening of the size distribution due to aggregation. Our findings confirm that this combined metric approach is valid and highly effective for monitoring the aggregation stability of inactivated EV71 particles.

This investigation demonstrated that pH plays a regulatory role in EV71 particle aggregation. The particles maintained stability between pH 7.0 and 7.5, while deviations from this range led to an increase in particle size. These findings indicate that controlling the pH within a physiological range during vaccine formulation and manufacturing can effectively prevent spontaneous aggregation of viral particles.

Temperature was also found to influence particle stability. Higher temperatures significantly enhanced viral particle aggregation. In addition to increasing Brownian motion and collision rates, elevated temperatures partially destabilize the tertiary structure of viral proteins, exposing hydrophobic regions that promote aggregation. This highlights the necessity of maintaining a continuous cold chain throughout the vaccine production and storage process. During inactivation of the EV71 virus, incubation at 37 °C is required, and even under these mild thermal conditions, the risk of aggregation remains considerable. Therefore, dynamic light scattering (DLS) is a valuable technique for real-time monitoring of particle size variations, allowing timely detection and control of aggregation induced during processing. Looking forward, the application of more advanced approaches, such as Advanced Kinetic Modeling (AKM), could integrate these and other accelerated stability data to predict long-term stability under recommended storage conditions (e.g., 2–8 °C) [29,30,31,32]. The implementation of such predictive models would help de-risk formulation development and provide a more scientifically robust justification for vaccine shelf-life.

Mechanical stress also had a pronounced influence on particle aggregation. The powerful shear forces generated by magnetic stirring induced significant aggregation, whereas agitation with a constant-temperature suspension mixture had no notable effect on the particle size. This divergence likely stems from the distinct modes of force application. The oscillatory motion of the suspension mixture produces mild mixing and periodic stress, which is sufficient for homogenization but insufficient to disrupt weak intermolecular interactions and initiate aggregation. In contrast, the localized high shear generated by magnetic stirring may compromise particle stability by triggering the disruption of secondary structure in the viral capsid proteins and the subsequent exposure of hydrophobic regions [33]. Magnetic stirring creates vortices and introduces air-liquid interfaces, where proteins can undergo selective orientation and cooperative unfolding [34]. This interface-induced denaturation is often more detrimental than shear stress alone. Furthermore, the physical contact and collisions between the magnetic stir bar and the container walls generate localized zones of extreme shear and compression, which are likely to induce protein denaturation. These observations provide essential guidance for optimizing process parameters to minimize exposure to such high-shear conditions. Notably, while the surfactant Tween 80 mitigated the shear stress from magnetic stirring, its ability to suppress aggregation was limited. This is attributed to the intense localized shear and compression at the solid-liquid interface between the stir bar and the container, which may directly denature viral particles—a mechanism that Tween 80 is ineffective at inhibiting.

Certain limitations should be acknowledged. The screening strategy primarily focused on the influence of individual stabilizers or simple buffer systems, whereas actual vaccine formulations consist of multiple components that may interact in complex ways. The synergistic or antagonistic interactions among different buffer salts and stabilizers were not systematically assessed. Hence, Design of Experiments approaches should be employed to formulate a stable vaccine composition capable of withstanding both thermal and mechanical stresses while evaluating multi-factor compatibility.

Moreover, various stress factors were examined separately in this study without integration into a complete formulation workflow. Future research should incorporate the identified stabilizer combinations into practical vaccine formulations and conduct comprehensive stability assessments under simulated manufacturing and storage conditions. Such studies would offer robust evidence supporting the real-world applicability and stability of the optimized formulation.

This study demonstrates the sensitivity of DLS in monitoring the physical aggregation of EV71 particles. To fully leverage its potential, future work should focus on establishing a quantitative correlation between DLS parameters and critical quality attributes directly linked to vaccine efficacy, such as antigen content measured by an antigenicity assay like ELISA and in vivo potency. Validating such a correlation would position DLS not merely as a physical stability indicator, but as a powerful predictive biomarker for the biological potency of inactivated EV71 vaccines. This would, in turn, streamline vaccine formulation development and enhance quality control paradigms.

## 5. Conclusions

The stability of the inactivated EV71 vaccine is dependent on the interplay between its complex physicochemical properties and environmental factors. This study revealed the effects of key factors (pH, temperature, and mechanical stress) on the aggregation behavior of viral particles at the particulate level and validated stabilization strategies targeting different stress sources. These findings provide direct guidance for the quality control of the EV71 vaccine and offer a valuable reference for developing and characterizing other similar viral particle vaccines.

## Data Availability

The methods for purification of EV71 vaccine bulks are confidential information of Wuhan Institute of Biological Products Co., Ltd., and they will not be shared. The data presented in this study are available on request from the corresponding author.

## Supplementary Materials

The following supporting information can be downloaded at: https://www.mdpi.com/article/10.3390/vaccines13121247/s1, Table S1: Detection results of standard particle size; Table S2: Detection results of particle size of EV71 bulk of different concentrations; Table S3: The particle size detection results of EV71 bulk repeated six times; Table S4: The particle size detection results of EV71 bulk for different days.
