# Supplementary material for: Inactivated Enterovirus 71 Particle Aggregation Stability: Dynamic Light Scattering Analysis and Stabilizer Identification
Source: Vaccines (Basel). 2025 Dec 15;13(12):1247. doi: 10.3390/vaccines13121247 (PMC12737540; doi:10.3390/vaccines13121247)
Supplement: Supplementary file 1 [file vaccines-13-01247-s001.zip › vaccines-4003836-supplementary.pdf]

Supplementary information

Inactivated Enterovirus 71 Particle Aggregation Stability: Dynamic Light Scattering Analysis and Stabilizer Identification

Anna Yang [1,2,3,4,†], Dongsheng Yang [1,2,3,4,†], Deqin Pang [1,2,3,4], Jie Yang [1,2,3,4], Wenhui Wang [1,2,3,4], Yaxin Du [1,2,3,4],
Xin Wan [1,2,3,4], Shengli Meng [1,2,3,4], Jing Guo [1,2,3,4,*] and Shuo Shen [1,2,3,4]

[1] Wuhan Institute of Biological Products Co. Ltd., No. 1 Huangjin Industrial Park Road,
Wuhan 430207, China; 2011302290006@whu.edu.cn (A.Y.); 18086496981@163.com (D.Y.);
pangdeqin@sinopharm.com (D.P.); yangjie107@sinopharm.com (J.Y.);
wangwenhui583@163.com (W.W.); duyaxin_duke@163.com (Y.D.);
wanxin3@sinopharm.com (X.W.); mengshengli@sinopharm.com (S.M.);
shenshuo1@sinopharm.com (S.S.)
[2] National Engineering Technology Research Center of Combined Vaccines, No. 1 Huangjin Industrial Park Road, Wuhan 430207, China
[3] State Key Laboratory of Novel Vaccines for Emerging Infectious Diseases, No. 1 Huangjin Industrial Park Road, Wuhan 430207, China
[4] Hubei Provincial Vaccines Technology Innovation Center, No. 1 Huangjin Industrial Park Road,
Wuhan 430207, China
* Correspondence: guojing27@sinopharm.com
† These authors contributed equally to this work.

Table S1. Detection results of standard particle size

Standard particle theoretical size (d.nm)	DLS detection size (d.nm)	Mean	SD
20±2	23.57	24.07	0.44
	24.35		
	24.30		
40±1	42.08	42.49	0.40
	42.51		
	42.88		

Table S2. Detection results of particle size of EV71 bulk of different concentrations

Protein concentration（μg/ml）	Size (d.nm)			Mean	SD	CV
	1	2	3			
10	35.89	36.15	34.64	35.56	0.81	2.27%
20	35.51	34.19	35.12	34.94	0.68	1.94%
40	36.83	36.76	35.79	36.46	0.58	1.59%
80	35.88	35.36	35.72	35.65	0.27	0.75%

Table S3. The particle size detection results of EV71 bulk repeated six times

Repetition times						Mean	SD	CV
1	2	3	4	5	6			

34.75	34.95	35.48	35.86	34.00	34.76	34.97	0.65	1.85%

Table S4. The particle size detection results of EV71 bulk for different days

**Precision**	Detection time			Mean	SD	CV
	Day 1	Day 2	Day 3			
Size (d.nm)	34.75	34.84	34.89	34.83	0.07	0.20%
